# Alterations in Ethanol-Induced Behaviors and Consumption in Knock-In Mice Expressing Ethanol-Resistant NMDA Receptors

**DOI:** 10.1371/journal.pone.0080541

**Published:** 2013-11-14

**Authors:** Carolina R. den Hartog, Jacob T. Beckley, Thetford C. Smothers, Daniel H. Lench, Zack L. Holseberg, Hleb Fedarovich, Meghin J. Gilstrap, Gregg E. Homanics, John J. Woodward

**Affiliations:** 1 Department of Neurosciences, Medical University of South Carolina, Charleston, South Carolina, United States of America; 2 Departments of Anesthesiology and Pharmacology & Chemical Biology, University of Pittsburgh, Pittsburgh, Pennsylvania, United States of America; The University of Tennessee HSC, Coll. Medicine, United States of America

## Abstract

Ethanol's action on the brain likely reflects altered function of key ion channels such as glutamatergic N-methyl-D-aspartate receptors (NMDARs). In this study, we determined how expression of a mutant GluN1 subunit (F639A) that reduces ethanol inhibition of NMDARs affects ethanol-induced behaviors in mice. Mice homozygous for the F639A allele died prematurely while heterozygous knock-in mice grew and bred normally. Ethanol (44 mM; ∼0.2 g/dl) significantly inhibited NMDA-mediated EPSCs in wild-type mice but had little effect on responses in knock-in mice. Knock-in mice had normal expression of GluN1 and GluN2B protein across different brain regions and a small reduction in levels of GluN2A in medial prefrontal cortex. Ethanol (0.75–2.0 g/kg; IP) increased locomotor activity in wild-type mice but had no effect on knock-in mice while MK-801 enhanced activity to the same extent in both groups. Ethanol (2.0 g/kg) reduced rotarod performance equally in both groups but knock-in mice recovered faster following a higher dose (2.5 g/kg). In the elevated zero maze, knock-in mice had a blunted anxiolytic response to ethanol (1.25 g/kg) as compared to wild-type animals. No differences were noted between wild-type and knock-in mice for ethanol-induced loss of righting reflex, sleep time, hypothermia or ethanol metabolism. Knock-in mice consumed less ethanol than wild-type mice during daily limited-access sessions but drank more in an intermittent 24 h access paradigm with no change in taste reactivity or conditioned taste aversion. Overall, these data support the hypothesis that NMDA receptors are important in regulating a specific constellation of effects following exposure to ethanol.

## Introduction

The consumption of alcoholic beverages produces a wide range of behavioral effects ranging from feelings of well being at lower doses to aversive or dysphoric effects at amounts that produce frank intoxication. Delineating the specific cellular and molecular mechanisms that underlie these acute effects has been difficult due to the diverse targets of alcohol action [1], [2] and the lack of alcohol antagonists that selectively reverse specific behaviors. Certain effects of ethanol are thought to arise from an interaction with key ion channels that regulate neuronal activity including glutamate-activated NMDARs that are widely acknowledged to be inhibited by doses of ethanol associated with behavioral impairment [3], [4].

NMDARs are heterotetramers composed of GluN1 and GluN2 (A–D) subunits and require glycine (or D-serine) and glutamate, respectively, for activation [5]. NMDAR subunits are arranged in a 1-2-1-2 configuration [6], [7] and differences in subunit expression yield receptors with distinct properties including differences in trafficking, post-translational modification, cellular distribution, and function [8]. NMDAR function is further influenced by endogenous modulators, such as polyamines, extracellular Zn^2+^ ions and protons that target the amino terminal domain [9].

While the sites and mechanisms of action of these allosteric modulators of NMDARs are well known, the precise way in which ethanol inhibits channel activity is unclear. Ethanol inhibition of NMDARs is non-competitive and voltage-independent [10]–[13], and persists even when large portions of the C-terminus are deleted [14]–[16]. At the single channel level, ethanol decreases mean open time and frequency of channel opening but does not affect single channel conductance [17] suggesting an interaction with sites involved in channel gating. Consistent with this idea, NMDA receptors made constitutively active by mutation of a highly conserved residue involved in gating (*SYTANLAAF*) lose much of their sensitivity to ethanol [18]. While the physical location of an ethanol site on the NMDAR is unknown, previous work by this lab and others showed that mutation of select residues in transmembrane domains (TMD) 3 and 4 of GluN1 and GluN2A subunits markedly reduce ethanol inhibition of the receptor [19]–[21].

In the present study, we generated knock-in mice that express a modified GluN1 subunit that reduces ethanol inhibition of NMDARs and test these animals for sensitivity to ethanol. The results of these studies show that reducing the ethanol inhibition of NMDAs produces behavior-specific differences following ethanol administration and alters ethanol consumption as compared to wild-type littermates.

## Materials and Methods

### Ethics Statement

Experiments were approved by Institutional Animal Care and Use Committee of MUSC and were conducted in accordance with National Institutes of Health guidelines with regard to the use of animals in research.

### Generation of Knock-In Mice

Gene targeting was similar to that described by Borghese *et al* [22]. Briefly, the GluN1 expression vector (kindly provided by R. Sprengel; see Single *et al* [23] for details) consisted of genomic Strain 129/Sv mouse DNA spanning exons 11–22 of the *Grin1* gene with a *lox*P-flanked neomycin phosphotransferase gene inserted into intron 18. Site-directed mutagenesis (QuikChange, Invitrogen) was used to replace the phenylalanine 639 codon in exon 16 (transmembrane domain 3) with one encoding alanine (F639A). The targeting vector contained ∼2.2 kb of 5′ and ∼8 kb of 3′ sequences relative to the Neo gene and was linearized with NotI and electroporated into R1 embryonic stem cells [24], [25] under previously described conditions [26]. G418 (Geneticin, 265 µg/ml; Invitrogen)-resistant embryonic stem cell clones were screened for gene targeting by Southern blot analysis of EcoRI digested DNA and hybridization with a 830 bp Avr-II-EcoRV probe derived from rat GluN1 cDNA [23]. Targeting was confirmed with additional digests/probes. The presence of the F639A mutation was confirmed by PCR/DNA sequence analysis. Target-positive ES cells were injected into C57BL/6J mouse blastocysts to generate germline competent chimeric animals. Knock-in mice were backcrossed to C57BL/6J mice for two generations (N2) prior to testing. Mice were genotyped by Southern blot or polymerase chain reaction from tail-derived DNA. Primers 5′-TTC ACA GAA GTG CGA TCT GG-3′ and 5′-AGG GGA GGC AAC ACT GTG GAC-3′ amplified a 466-base pair fragment from the wild-type allele. Primers 5′-CTT GGG TGG AGA GGC TAT TC-3′ and 5′-AGG TGA GAT GAC AGG AGA TC-3′ amplified a 280-base pair fragment from the knock-in allele.

After weaning, mice were housed with ad libitum access to rodent chow and water with 12-h light/dark cycles (lights on at 6:00 AM unless otherwise specified). All mice used for behavioral and electrophysiological experiments were male and at least 8 weeks old.

### Preparation of Recombinant Cultures and Brain Slices

Studies using human embryonic kidney 293 (HEK293) cells (American Type Culture Collection, Manassas, VA) were performed as previously described [21] and were transfected with equal amounts (typically 1 µg each) of cDNA plasmids encoding various NMDAR subunits and enhanced GFP using Lipofectamine 2000 (Invitrogen). Dissociated hippocampal cultures for electrophysiological recordings were prepared from hippocampi isolated from embryonic day 18 mice as described previously [27]. Cultures were incubated at 37°C (95% CO_2_/5% O_2_) on poly-L-lysine-coated 35 mm culture dishes for up to 3 weeks and the feeding media was changed at least once a week. Acute brain slices were prepared as described in [28]. Briefly, mice (12 weeks or older) were rapidly decapitated, brains were removed and placed in an ice-cold sucrose solution that contained (in mM): sucrose (200), KCl (1.9), NaH_2_PO_4_ (1.4), CaCl_2_ (0.5), MgCl_2_ (6), glucose (10), ascorbic acid (0.4) and NaHCO_3_ (25); osmolarity 310–320 mOsm, bubbled with 95% O_2_/5% CO_2_ to maintain physiological pH. Coronal sections containing the prefrontal cortex (PFC) were cut into 300 µm slices using a Leica VT1000 vibrating microtome (Buffalo Gove, IL) with a double-walled chamber through which cooled (2–4°C) solution was circulated. Slices were collected and transferred to a warmed (32–34°C) chamber containing a carbogen-bubbled aCSF solution containing (in mM): NaCl (125), KCl (2.5), NaH_2_PO_4_ (1.4), CaCl_2_ (2), MgCl_2_ (1.3), glucose (10), ascorbic acid (0.4) and NaHCO_3_ (25); osmolarity 310–320 mOsm. Slices were warmed for 30 min and then kept at room temperature under carbogen bubbling for at least 45 minutes before beginning recordings. Following incubation, slices were transferred to a recording chamber and perfused with aCSF at 2 ml/min. Experiments were conducted at a bath temperature of 32°C controlled by in-line and bath heaters (Warner Instruments, Hamden, CT).

### Electrophysiology

Currents in cultured hippocampal neurons and HEK293 cells transfected with various NMDAR subunits were measured using whole-cell patch-clamp electrophysiology as described previously [21], [27]. For slice recordings, neurons were visually identified under infrared light using an Olympus BX51WI microscope with Dodt gradient contrast imaging (Luigs and Neumann, Ratingen, Germany). Whole cell patch-clamp recordings were performed in deep-layers of the prelimbic mPFC and targeted large, pyramidal-shaped neurons with prominent apical dendrites. The recording aCSF was supplemented with 100 µM picrotoxin (Tocris Bioscience, Ellsville, MO) to block GABA_A_ receptors and 10 µM 2,3-dioxo-6-nitro-1,2,3,4-tetrahydrobenzo(f)quinoxaline-7-sulfonamide (NBQX; Abcam Biochemicals, Cambridge, MA) to block AMPA receptors. Recording pipettes (resistance of 1.5–3.5 MΩ) were filled with internal solution containing: (in mM): CsCl (120), HEPES (10), MgCl_2_ (2), EGTA (1), Na_2_ATP (2), NaGTP (0.3); osmolarity 295 mOsm, pH = 7.3. After gigaohm seal and breakthrough at −70 mV, cells were slowly ramped to +40 mV and NMDA excitatory postsynaptic currents (EPSCs) were evoked using a tungsten concentric bipolar electrode (0.1 ms pulse width) at a setting that elicited reliable, sub-maximal responses. In some experiments, changes in holding current were monitored during bath application of 5 µM NMDA (Sigma-Aldrich, Saint Louis, MO) to assess expression of synaptic and extrasynaptic NMDA induced currents. In all experiments, series resistance (R_s_) was monitored throughout the recording and an experiment was discontinued if R_s_ exceeded 25 MΩ or changed more than 25%. Data were acquired using an Axon MultiClamp 700B amplifier (Molecular Devices, Union City, CA) and an ITC-18 digital interface (HEKA Instruments, Bellmore, NY) controlled by AxographX software (Axograph Scientific, Sydney, NSW, Australia). Recordings were filtered at 4 kHZ, acquired at 10 kHz and analyzed offline using AxographX software.

### Western Blotting

NMDAR subunit expression in mice was analyzed by western blotting as previously described by Pava *et al* [29]. Briefly, animals were rapidly euthanized by decapitation, and brains were immediately immersed for 1–2 min in ice-cold dissection buffer containing (in mM): sucrose (200), KCl (1.9), MgCl_2_ (6), CaCl_2_ (0.5), glucose (10), ascorbic (0.4) acid, HEPES (25), pH 7.3 with KOH. Brains were sectioned into 1–2 mm thick coronal slices using an adult mouse brain matrix (ASI Instruments, Warren, MI) and crude membrane fractions were isolated from 5 brain regions (medial prefrontal cortex, mPFC; dorsal striatum, DS; nucleus accumbens, NAcc; hippocampus, HC; and basolateral amygdala, BLA) from each mouse. Antibodies used in these studies were GluN1 (BD Pharmingen, Franklin Lakes, NJ), GluN2A (Millipore, Billerica, MA) and GluN2B (NeuroMab, Antibodies Inc., & UC Davis, Davis, CA). The band corresponding to appropriate subunit was quantified by mean optical density using computer-assisted densitometry with ImageJ v1.41 (National Institutes of Health, USA). Data are expressed as the percent of the wild-type control run simultaneously on each blot.

### Locomotor Activity

Locomotor activity in mice was measured using activity chambers (40×40×30 cm) (Digiscan Activity Monitors, Omnitech Electronics, Inc., Columbus, OH) contained within sound-attenuating boxes and were interfaced with a computer running Versamax software (Accuscan Instruments, Inc., Columbus, OH). Activity was quantified by the number of photobeam breaks during the test session and was converted to total distance traveled (cm). All locomotor activity testing was done one week apart and mice were injected 5 minutes before being placed in the activity chamber for 10 min. Baseline locomotor activity was first tested in all mice following treatment with saline. Mice were then tested weekly following treatment with saline or different concentrations of ethanol (0.75, 1.5, or 3.0 g/kg; IP) in a latin-square design so that each mouse received all doses. In a separate cohort of animals, locomotor activity was monitored by placing mice inside an opaque box (40×40×40 cm) 30 min following injection with either saline or the selective NMDA antagonist MK-801 (0.3 mg/kg; IP). Total distance traveled (cm) over a 10 min test period was measured using a video tracking system (ANYmaze, Stoelting Co., Wood Dale, IL). In all cases, mice were given one hour to acclimate to transport into the testing room and all activity chambers were cleaned in between animals.

### Loss of Righting Reflex/Sleep Time/Hypothermia

The sedative/hypnotic effect of ethanol was determined by measuring the latency and duration of the loss of righting reflex (LORR). Mice were injected with ethanol (4.0 g/kg; IP) and the latency to LORR was measured as the time from injection until mice were unable to right themselves within 30 s of being placed in a supine position. Sleep time was measured from the onset of the LORR until the time that mice regained their ability to right themselves twice within a 30 s period. In a separate group of mice, animals were administered 3.5 g/kg ethanol (IP) and body temperature was monitored using a rectal probe.

### Motor Coordination

One day prior to ethanol testing, mice were trained to remain on a fixed-speed (5 rpm) rotarod (Ugo Basile, Comerio, Italy) for a period of 60 s without falling. The next day, animals were re-trained, injected with either 2.0 or 2.5 g/kg ethanol (IP) and then placed on the rotarod. The time to fall was recorded and mice were placed back on the rotarod at various times until they remained on the rotarod for 60 s. To avoid ethanol-dependent learning effects, each mouse received only a single dose of ethanol.

### Anxiety Testing

An elevated zero maze (Med Associates; St Albans, VT) was used to determine general levels of anxiety in mice. Mice were first habituated to transport and handling for 3 days and then tested on the maze under dim room lighting. The study was run in two separate, naïve cohorts tested one week apart and counter-balanced for genotype and treatment. On the test day, mice were given 1 hour to acclimate to the testing room and then injected with either saline or ethanol (1.25 g/kg; IP). Five minutes later, they were placed in the center of one of the closed arms of the elevated zero maze and were monitored for 5 min using a video tracking system (ANYmaze, Stoelting Co., Wood Dale, IL). Measured parameters included time spent in closed and open arms, number of entries into closed and open arms and total distance traveled.

### Ethanol Metabolism

Mice were injected with ethanol (4.0 g/kg; IP) and blood samples were taken from the retro-orbital sinus at 30, 120 and 240 min post-injection. The study was repeated a week later and samples were taken at 60, 120 and 180 min post-injection. Blood ethanol concentration (BEC) values were determined as described by Pava *et al* [29] and were expressed as milligram of ethanol per deciliter of blood.

### Drinking Studies

A series of studies using different drinking paradigms was used to assess the effect of the F639A mutation on ethanol consumption, tastant preference and conditioned taste aversion. In all studies, mice were individually housed for at least 1 week prior to initiating the drinking study and food was provided *ad libitum* at all times. Where appropriate, the placement of the drinking bottles was alternated for each session to control for side preferences and mice were weighed weekly. Sham cages had drinking tubes but no mice to account for accidental spillage or loss of fluid. Unless otherwise noted, separate cohorts of age-matched F639A Het mice and wild-type littermates were used in each study.

### Two-Bottle Choice Limited Access

One-half hour prior to lights off, home cage water bottles were replaced with two drinking tubes containing either 15% (v/v with water) ethanol or water. Drinking tubes were weighed immediately before and 2 h after each daily drinking session and the difference in volume was converted to g/kg consumed. At all other times, mice had free access to home cage water bottles.

### One-Bottle Limited Access Drinking in the Dark (DID)

Three h after lights off, the water bottle in each cage was replaced with a bottle containing ethanol (20% v/v) for either 2 h (first 3 days) or 4 h (4^th^ day). This pattern was repeated every 4 days and consumption was converted to g/kg. Following each test session, the ethanol bottle was replaced with home cage water bottles.

### Two-Bottle Choice 24 h Intermittent Access (IA)

Mice were given access to two bottles of water for 24 h and on the next day, bottles were replaced with ones containing either ethanol or water. This pattern of access to ethanol was alternated every 24 h. The concentration of ethanol presented for the first drinking session was 3% and was increased during successive sessions to 6%, 10%, 15% and then 20% for the remainder of the study. In a similar study, mice were given intermittent (every other day) 24 h access to either two bottles of water or water and one containing 3% ethanol sweetened with saccharin (0.2%). The ethanol concentration (all with 0.2% saccharin) was ramped up during successive sessions to 6%, 10%, 15%, 20% and finally 40%.

### Non-alcohol Tastant Testing

Preference for sweet and bitter solutions was tested by presenting mice with a bottle of water and a bottle of a tastant solution each day for four days. Tastants included 0.033, 0.066, and 1% saccharin (2,3-Dihydro-3-oxobenzisosulfonazole sodium salt; Sigma-Aldrich, St. Louis, MO), 0.03 and 0.06 mM quinine (prepared from the hemisulfate salt monohydrate; Sigma-Aldrich, St. Louis, MO), and 1.7 and 4.25% sucrose (Sucrose, Fisher Scientific) in that order. Mice were tested for 4 days with each concentration of a tastant and then given two weeks off with *ad libitum* home cage water bottle access before receiving the next tastant.

### Conditioned Taste Aversion

Prior to testing, mice were water restricted (2 h of water per day) over a 7-day period to ensure robust consumption during the test period. The following day, mice were given 1 h access to a saccharin solution (0.15% (w/v) sodium saccharin in tap water) followed immediately by an injection of either saline or ethanol (1.25 or 2.5 g/kg; IP). Saccharin consumption was measured 24 h later during a 1 h test period and the reduction in saccharin intake was used as a measure of conditioned taste aversion. To prevent dehydration on test days, mice were given 30 min access to water 5 h post-injection. The water restriction schedule was maintained on intervening non-injection days.

### Statistical Analysis

Data were reported as the mean ±S.E.M. and analyzed using Prism (GraphPad Software Inc., San Diego, CA). Analysis of variance (two-way ANOVA with Bonferroni's *post hoc* tests) with or without repeated measures (RM) and Student's *t* test were carried out to evaluate differences between groups. To evaluate differences within groups, analysis of variance (one-way ANOVA with Bonferroni's *post hoc* tests) was carried out. A linear mixed model ANOVA with *post hoc* Bonferroni testing was used to assess differences in drinking across days between groups.

## Results

### Characterization of Mutant Mice

**Figure 1:**
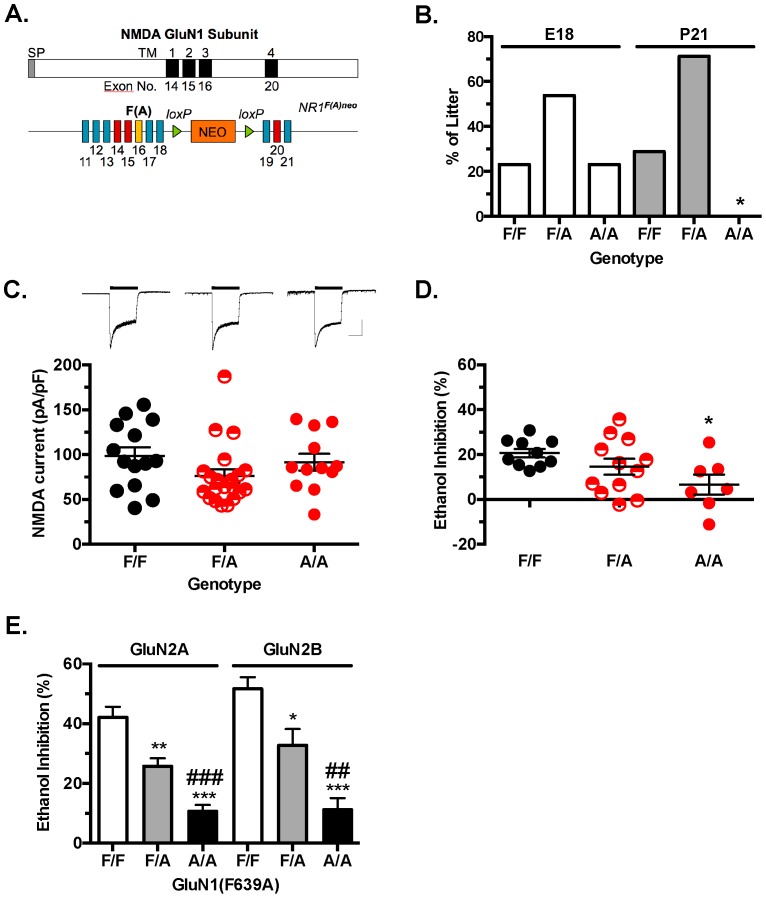
Targeted point mutation (F639A) in the GluN1 subunit decreases ethanol sensitivity of NMDA receptors. (*A*), Top: Schematic of GluN1 protein with transmembrane domains (*solid bars*) and corresponding exons. Bottom: Gene construct used to generate the F639A mice. F(A) is site of mutation within exon 16. NEO cassette flanked by *loxp* sites is between exons 18 and 19. (*B*), Percent of wild-type (F/F), heterozygous (F/A), and homozygous (A/A) F639A mice alive at embryonic day 18 or post-natal day 21. Symbol: (*) no surviving mice. (*C*), Top panel: Sample traces from 14-day old primary hippocampal cultures during (*black bar*) application of 50 µM NMDA/10 µM glycine. Scale bars: y-axis, 2000 pA; x-axis, 2.5 ms. Bottom panel: Mean amplitude of NMDA evoked currents in cultures from wild-type (F/F, n = 14), heterozygous (F/A, n = 21) and homozygous (A/A, n = 12) F639A mice. (*D*), Ethanol inhibition from 14-day old primary hippocampal cultures. Percent inhibition of steady state current by 100 mM ethanol from wild-type (F/F, n = 10), heterozygous (F/A, n = 12) and homozygous (A/A, n = 7) F639A mice. Symbol (*): value significantly different from wild-type (* *p*<0.05; one-way ANOVA, Dunnett's *post hoc* test). (*E*), Ethanol inhibition of recombinant wild-type and mutant NMDA receptors expressed in HEK293 cells. Data represent percent inhibition by 100 mM ethanol in cells expressing GluN1 or GluN1(F639A) with either GluN2A (F/F, n = 5; F/A, n = 14; A/A, n = 10) or GluN2B subunits (F/F, n = 6; F/A, n = 8; A/A, n = 9). Symbols: (*) significantly different from wild-type (* *p*<0.05, ** *p*<0.01, *** *p*<0.001; one-way ANOVA, Bonferroni's *post hoc* test); (**#**) significantly different from F639A Het (**##** *p*<0.01, **###** *p*<0.001; one-way ANOVA, Bonferroni's *post hoc* test).

Gene targeting was used to generate mice heterozygous for the mutant F639A *Grin1* allele containing a floxed Neo selection cassette (F639A Het; Figure 1A). Adult F639A Het males and females were bred together to generate wild-type (F/F), heterozygous (F/A), and homozygous (A/A) offspring. Genotyping of animals at weaning (post-natal day 21) revealed only wild-type or heterozygous mice while those genotyped at embryonic day 18 (E18) showed the expected frequency of wild-type, heterozygous and homozygous offspring (Figure 1B). Previous studies show that global GluN1 knockout (KO) mice die within the first post-natal day [30] suggesting that homozygous F639A Het mice may express non-functional NMDA receptors. To test this, primary hippocampal neurons were isolated from individual pups (E18) generated from Het x Het breeding pairs and were maintained in cultures for 2–3 weeks. In 14-day old cultures, local application of NMDA to patched-clamped neurons evoked robust currents in all genotypes (Figure 1C) and there were no significant differences in mean amplitude of NMDA evoked currents across groups (F_(2,44)_ = 1 92, *p* = 0.16). As a strain of NMDA hypomorph mice are viable despite expressing only 5% of the normal GluN1 subunit [31], these results suggest that it is unlikely that the neonatal lethality of homozygous F639A mutants results from insufficient expression of functional NMDA receptors. Ethanol (100 mM) inhibited NMDA currents in neurons from cultures prepared from wild-type (F/F) GluN1 mice by approximately 20% (Figure 1D) while having significantly less effect (∼7% inhibition) on currents in neurons from mice homozygous (A/A) for the mutant allele (one-way ANOVA: main effect of genotype, F_(2,26)_ = 3.690, *p*<0.05). Ethanol inhibition of NMDA currents in neurons prepared from heterozygous animals (F/A) showed a range of ethanol inhibition that overlapped that observed for wild-type and homozygous mice suggesting that a single mutant allele can reduce ethanol inhibition of NMDA currents. To further examine whether co-expression of wild-type and mutant GluN1 subunits affects ethanol inhibition of NMDARs of both GluN2A and GluN2B containing receptors, HEK293 cells were transfected with different combinations of wild-type and/or mutant NMDAR subunits and tested for ethanol inhibition. Ethanol (100 mM) significantly inhibited glutamate-activated currents in cells expressing the wild-type GluN1 subunit and either the GluN2A or GluN2B subunit (Figure 1E). Replacing the wild-type GluN1 subunit with the GluN1(F639A) mutant significantly reduced the effect of ethanol and cells transfected with equal amounts of wild-type and mutant (F639A) GluN1 cDNAs showed an intermediate sensitivity to ethanol (one-way ANOVA: main effect of genotype, GluN2A, F_(2,26)_ = 22.63, *p*<0.0001; GluN2B, F_(2,20)_ = 18.93, *p*<0.0001). Similar to the results obtained with the primary cultures, these results suggest that NMDA responses in mice heterozygous for both wild-type and GluN1(F639A) alleles would be expected to show reduced sensitivity to ethanol especially at lower concentrations that are associated with behavioral intoxication (∼22–66 mM; 100–300 mg/dl blood ethanol concentration).

Based on these findings, all further experiments were conducted with wild-type and heterozygous littermates generated from Het x Het breeding pairs. There were no differences seen in body weight or growth rate between groups of wild-type and F639A Het mice used in the study. For example, in a typical cohort, average body weights for wild-type and mutant male mice at 11–12 weeks of age were 27.36±0.57 g (n = 20) and 29.44±1.13 g (n = 18) respectively.

### NMDA Responses to Ethanol in mPFC Slices from F639A Het mice

**Figure 2:**
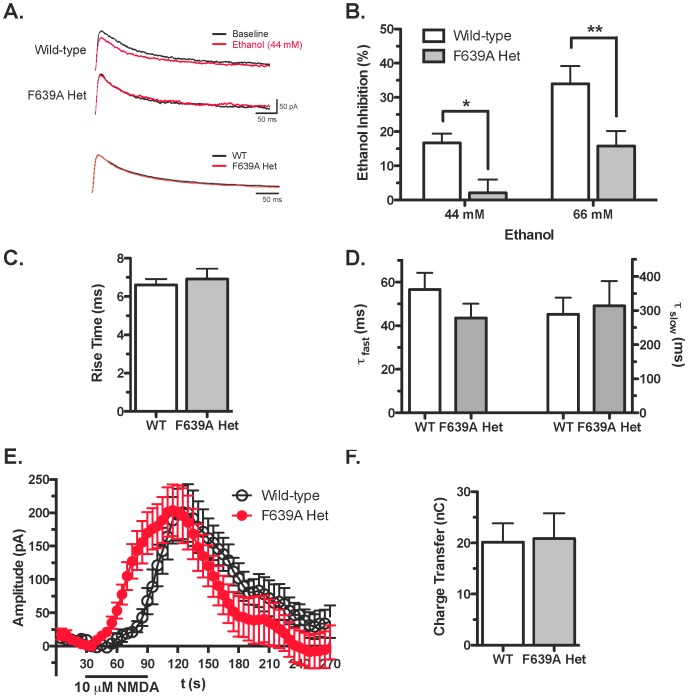
GluN1(F639A) mutation alters ethanol inhibition of NMDA-mediated currents in adult mice. (*A*), Top: Sample traces of electrically evoked NMDA EPSCs in mPFC neurons from wild-type and F639A Het mice at baseline (*black*) and during exposure to 44 mM ethanol (*red*). Bottom: Control NMDA EPSCs from wild-type and F639A Het mice normalized by amplitude. (*B*), Summary of ethanol inhibition of NMDA-mediated EPSCs in neurons from wild-type (44 mM, n = 10; 66 mM, n = 7) and F639A Het mice (44 mM, n = 9; 66 mM, n = 10). Data are percent of control (mean ±SEM). Symbol (*): value significantly different from wild-type (* *p*<0.05; ** *p*<0.01; two-way ANOVA, Bonferroni's *post hoc* test). (*C*), Rise time (mean ±SEM) of NMDA-mediated EPSCs in wild-type (n = 7) and F639A Het mice (n = 9). (*D*), Mean values (±SEM) for fast (left) and slow (right) decay time constants of NMDA-mediated EPSCs from wild-type (fast, n = 7; slow, n = 9) and F639A Hets (fast, n = 7; slow, n = 9). (*E*), Change in holding current of mPFC neurons from wild-type and F639A Het mice before, during, and after bath application of 5 µM NMDA (n = 7–8 for each group). Values are mean ±SEM. (*F*), Total charge transfer through NMDA receptors in wild-type and F639A Het mice (n = 7–8 for each group). Values are mean ±SEM.

**Figure 3:**
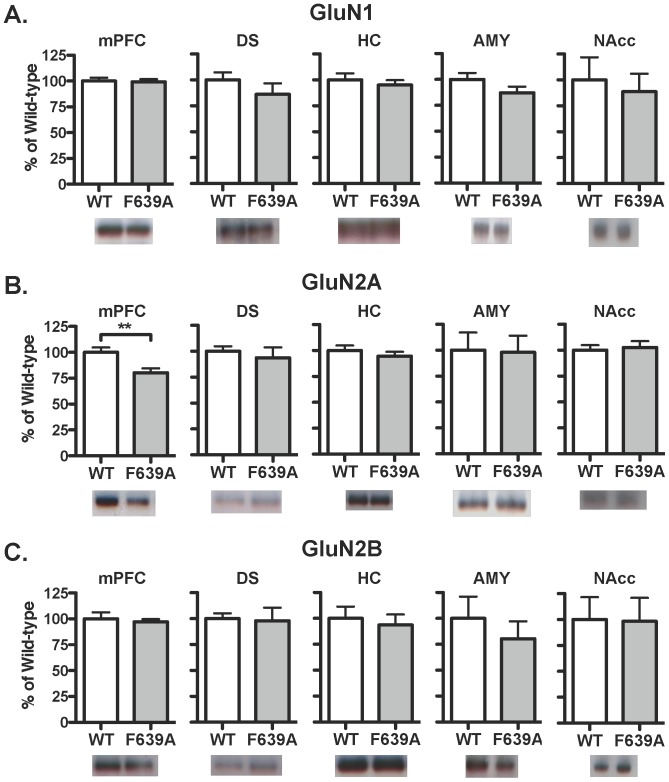
Expression of NMDA receptor subunits in wild-type and F639A Het mice (n = 4–5 for each group). Panels show immunoblot analysis of GluN1 (*A*), GluN2A (*B*), and GluN2B (*C*) from crude membrane fractions prepared from select brain regions. Data are percent of wild-type control (mean ±SEM). Abbreviations: *mPFC*, medial pre-frontal cortex; *DS*, dorsal striatum; *HC*, hippocampus; *AMY*, amygdala; and *NAcc*, nucleus accumbens. Symbol (*): value significantly different from control (** *p*<0.01, unpaired *t*-test).

Whole-cell patch-clamp electrophysiology was used to examine the functional status and ethanol sensitivity of NMDA receptors in brain slices prepared from adult wild-type and heterozygous F639A mice. Neurons were held at +40 mV in normal magnesium containing ACSF and NMDA-mediated synaptic EPSCs were evoked in layer V mPFC pyramidal neurons in the absence and presence of different concentrations of ethanol. As shown in Figure 2, ethanol produced greater inhibition of NMDA EPSCs in wild-type mice compared to mutant mice (two-way ANOVA: main effect of genotype, F_(1,32)_ = 16.56, p<0.001 and ethanol treatment, F_(1,32)_ = 14.90, p<0.001). For example, 44 mM ethanol (∼200 mg/dl BEC) reduced the amplitude of NMDA EPSCs in wild-type mice by approximately 15% while EPSCs from mutant mice were largely unaffected (Figure 2B). At 66 mM ethanol, ethanol inhibited NMDA EPSCs from wild-type mice by approximately 35% while currents in mutant mice were reduced by ∼15%. There were no genotype specific differences in EPSC rise time (*t*_(14)_ = 0.45, ns; Figure 2C) or decay kinetics (T_(fast)_−*t*_(12)_ = 1.31, ns; T_ (slow)_−*t*_(12)_ = 0.26, ns; Figure 2D) measured under control conditions. To examine total (synaptic plus extrasynaptic) NMDA receptor function, mPFC neurons were voltage-clamped at +40 mV and changes in holding current were monitored during bath application of NMDA (10 uM). NMDA induced a reproducible increase in holding current and the magnitude of this effect was not different between the two genotypes (*t*_(13)_ = 0.06, ns; Figure 2E). Although the amplitudes and total charge transfer in response to NMDA were similar between groups (Figure 2F), currents in neurons from heterozygous mice rose more quickly during bath-applied NMDA than those from wild-type mice (mixed ANOVA: main effect of time, F_(53,689)_ = 24.63, *p*<0.001; and a significant interaction, F_(53,689)_ = 3.831, *p*<0.001; Figure 2E). To examine expression of NMDAR subunits in adult animals, western blotting was performed on brain tissue isolated from adult wild-type and mutant mice. As shown in Figure 3, there were no changes in the expression of the GluN1 or GluN2B subunits between wild-type and F639A Het mice in dorsal striatum, hippocampus, amygdala and nucleus accumbens. Levels of GluN2A were also similar between wild-type mice and F639A Het mice in all regions except for the mPFC where there was a slight reduction (∼20%) noted for F639A Het mice (*t*_(8)_ = 3.2, *p*<0.05; Figure 3C).

### Motor Effects of Ethanol

**Figure 4:**
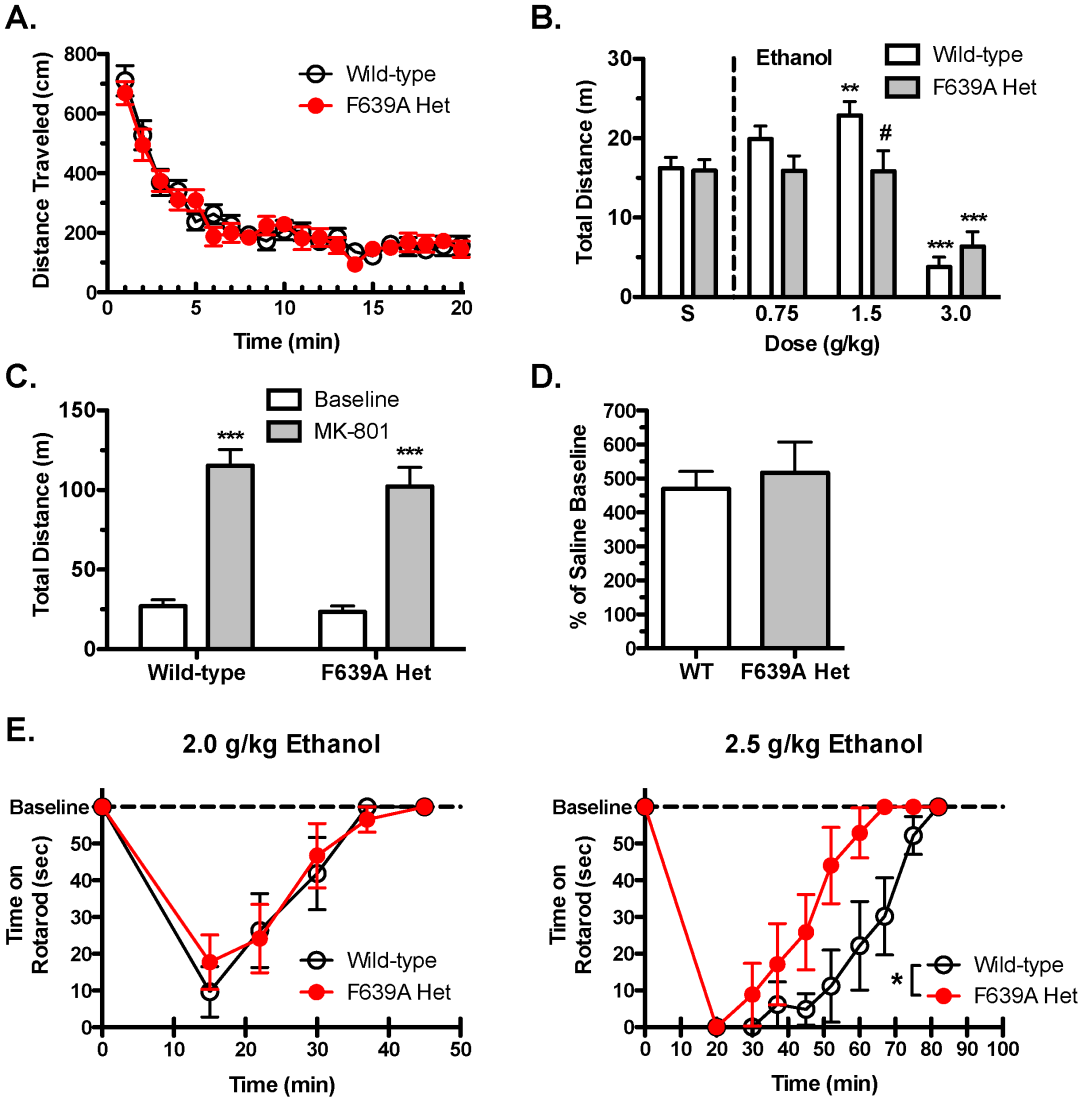
Locomotor stimulating effects of ethanol are blunted in F639A Het mice. (*A*), Baseline spontaneous locomotor activity in saline-treated wild-type and F639A Het mice (n = 15 for each group). Distance (mean ±SEM) traveled shown in 1 min-bins. (*B*), Summary plot showing total distance (mean ±SEM) traveled by mice during a 10 min period following injection of either saline or ethanol. Symbol (*): value significantly different from saline (** *p*<0.01, *** *p*<0.001, two-way RM ANOVA, Bonferroni's *post hoc* test); (**#**) value significantly different from wild-type (**#** *p*<0.05, two-way RM ANOVA, Bonferroni's *post hoc* test). (*C*), Total distance (mean ±SEM) traveled by wild-type and F639A Het mice during a 10 min test period after treatment with saline (baseline) or MK-801 (0.3 mg/kg) (n = 7 for each group). Symbol (*): value significantly different from saline (*** *p*<0.001, two-way RM ANOVA, Bonferroni's *post hoc* test). (*D*), Total distance (mean ±SEM) traveled under acute MK-801 treatment shown as percent of baseline (saline) treatment. (*E*), Time (mean ±SEM) spent on a fixed-speed rotarod following injection of 2.0 g/kg (n = 6–7 for each group) or 2.5 g/kg (n = 6–7 for each group) ethanol in wild-type and F639A Het mice. Symbol (*): value significantly different from wild-type (* *p*<0.05, two-way RM ANOVA, Bonferroni's *post hoc* test).

Wild-type and F639A Het mice were tested in locomotor chambers following injection with saline or ethanol. After saline treatment, both groups of mice showed similar spontaneous locomotor activity when placed in the novel environment (*p* = 0.65) that decreased over time (Figure 4A). Following injection of ethanol, wild-type mice showed a biphasic response with lower doses (0.75–2.0 g/kg) increasing activity and the highest dose (3.0 g/kg) decreasing locomotion. In contrast, F639A Het mice showed no increase in activity following injection with 0.75–2.0 g/kg ethanol while the highest dose reduced distance travelled (two-way RM ANOVA: main effect of dose, F_(3,117)_ = 30.15, *p*<0.0001; dose × genotype interaction, F_(3,117)_ = 3.02, *p*<0.05).

To test whether the lack of ethanol-induced stimulation of locomotor activity in F639A Het mice generalized to other NMDA antagonists, mice were tested following administration of MK-801. As reported above, there were no differences in locomotor activity between groups following treatment with saline. MK-801 induced a robust and highly significant increase in locomotor activity in both F639A Het and wild-type mice (two-way RM ANOVA: main effect of treatment, F_(1,12)_ = 119.1, *p*<0.05; Figure 4C), and the magnitude of this effect did not differ between the genotypes (Figure 4D).

The motor incoordinating effects of moderate doses of ethanol (2.0 and 2.5 g/kg) were measured using the rotarod test. Acute administration of 2.0 g/kg ethanol produced motor ataxia in both groups of mice illustrated by a significant reduction in time spent on the rotarod (two-way RM ANOVA: effect of time, F_(4,44)_ = 25.44, *p*<0.0001; Figure 4E). Over time, performance improved and both groups regained normal function approximately 40 min following the initial injection of ethanol. Mice in both groups showed more sustained impairment in rotarod activity when injected with a slightly higher dose (2.5 g/kg) of ethanol (two-way RM ANOVA: effect of time, F_(8,88)_ = 28.19, *p*<0.0001; Figure 4F). However, F639A Het mice recovered motor function significantly faster than wild-type mice at the 2.5 g/kg dose (two-way RM ANOVA: main effect of genotype, F_(1,88)_ = 6.57, *p*<0.05; time × genotype interaction, F_(8,88)_ = 2.44, *p*<0.05).

### Ethanol-induced LORR, Hypothermia and Blood Ethanol Metabolism

**Figure 5:**
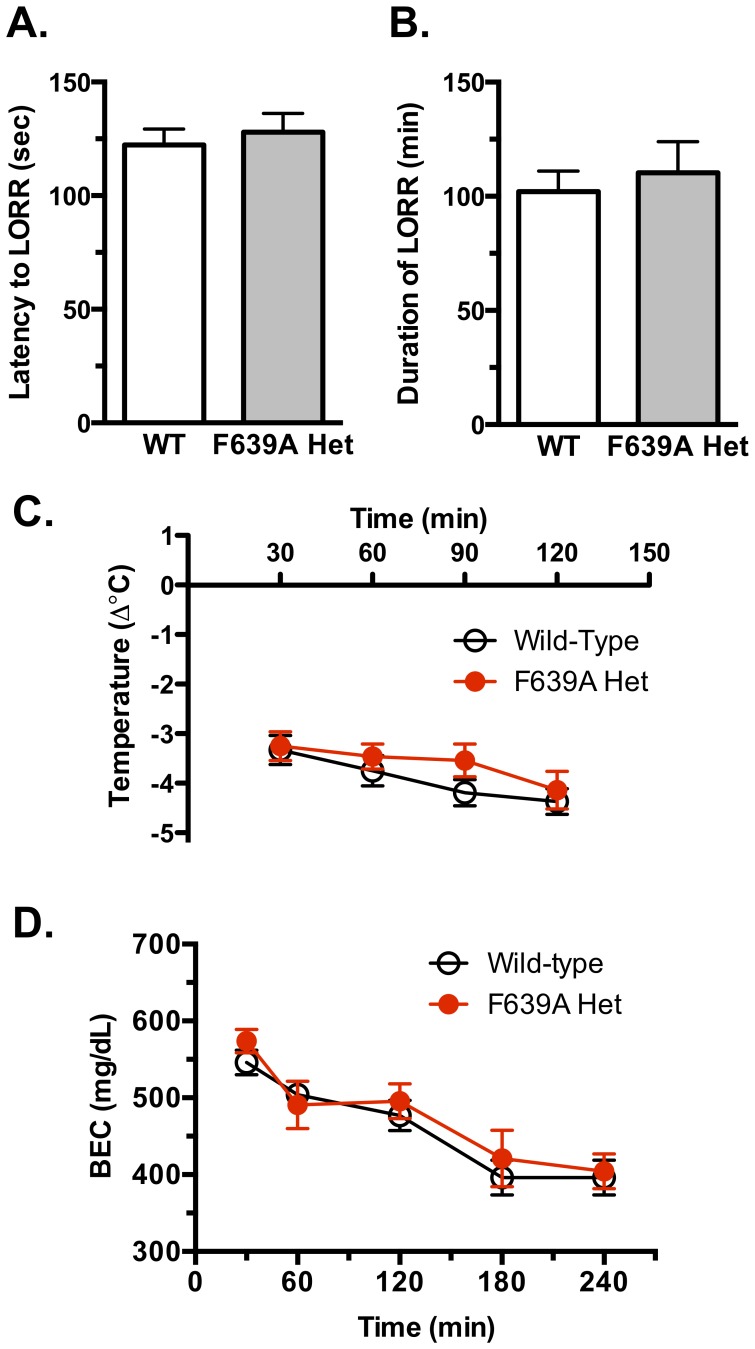
Hypnotic and hypothermic effects of high doses of ethanol. Latency to lose righting reflex (LORR; *A*) and duration of LORR (*B*) following a 4.0 g/kg injection of ethanol in wild-type and F639A Het mice (n = 17–18 for each group). Values are mean ±SEM. (*C*), Change in body temperature following a 3.5 g/kg injection of ethanol in wild-type and F639A Het mice (n = 10 for each group). Values are mean ±SEM. (*D*), Rate of blood ethanol metabolism between wild-type and F639A Het mice. Blood ethanol concentration (mean ±SEM) measured over time following injection with 4.0 g/kg of ethanol (n = 7 for each group).

To measure the sedative/hypnotic effects of ethanol, wild-type and F639A Het mice were injected with 4.0 g/kg ethanol and onset latency and duration of LORR were recorded. Both groups showed similar latency to onset of LORR that occurred within approximately 2 min following injection (Figure 5A). There was also no significant difference in duration of LORR between (Figure 5B) F639A Het mice (102.1±8.98 min, n = 17) compared to wild-type mice (110.3±13.63 min, n = 18).

As sensitivity to the sedative and rewarding effects of ethanol in rodents has been correlated with initial sensitivity to ethanol's hypothermic effects [32], rectal temperature was monitored in wild-type and Het mice following an acute injection of ethanol (3.5 g/kg ethanol). There were no differences in baseline body temperature between wild-type (38.18±0.15°C) and F639A Het mice (38.17±0.18°C) prior to ethanol treatment. Both groups of mice showed significant hypothermia (∼3°C) within 30 min of the ethanol challenge and the magnitude of this effect was not different between groups (Figure 5C).

The rate of ethanol clearance was measured in both groups to determine whether differences in ethanol metabolism could account for any of the genotypic-specific differences in ethanol-induced behaviors. Thirty minutes following injection of mice with 4 g/kg ethanol, blood ethanol concentrations in both groups were ∼550–570 mg/dl and concentrations declined slowly over the next 4 h. There were no differences in the rate of ethanol clearance between wild-type and F639A Het mice (Figure 5D).

### Anxiolytic Effects of Ethanol

**Figure 6:**
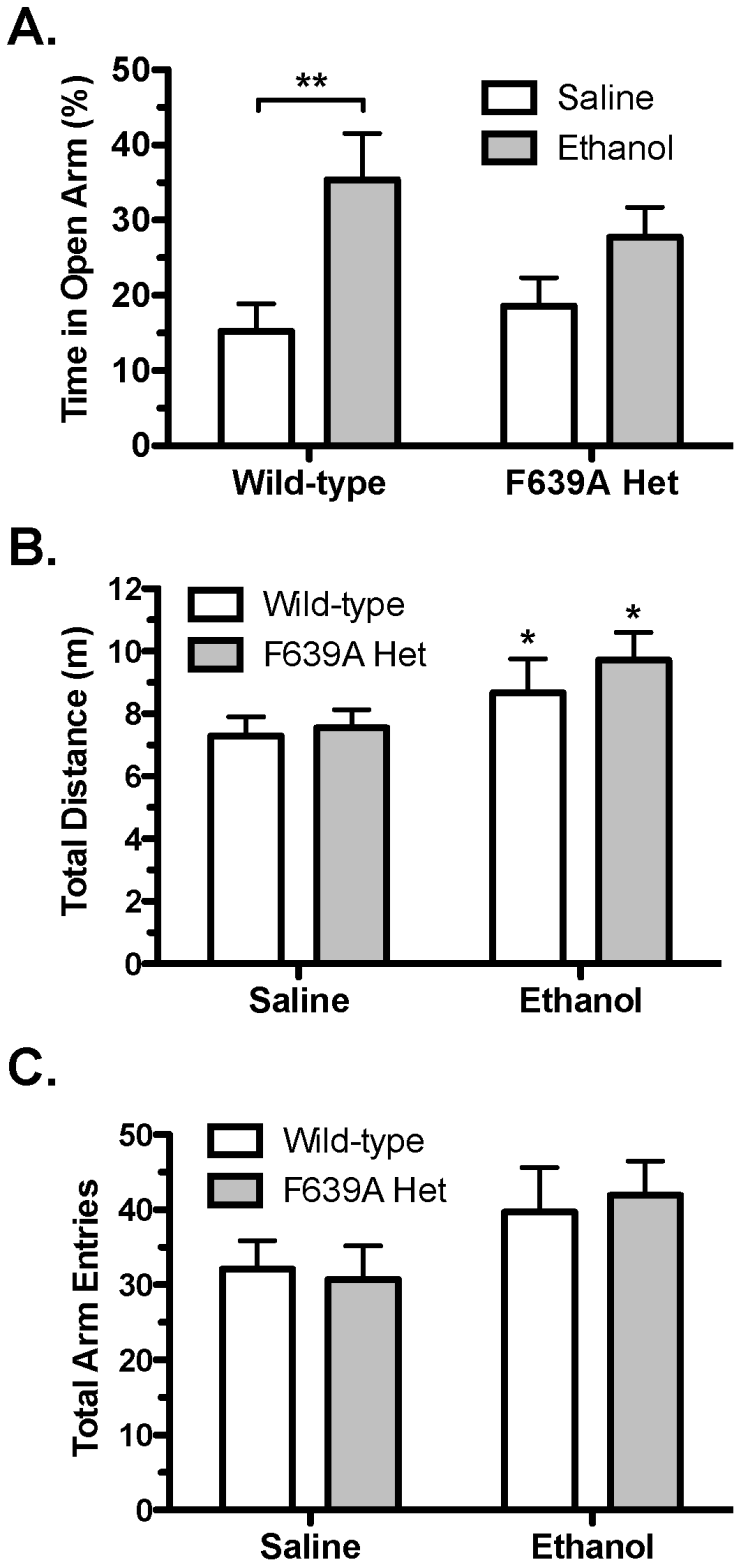
Anxiolytic response to ethanol is blunted in F639A Het mice. (*A*), Percent of time (mean ±SEM) spent in the open arms of an elevated zero maze following injection of saline or 1.25 g/kg ethanol in F639A Het and wildtype mice (n = 10 for each group). Symbol (*): value significantly different from saline (** p<0.01, two-way ANOVA, Bonferroni's *post hoc* test). (*B*), Total distance traveled and (*C*), total number of arm entries on the elevated zero maze. Symbol (*): value significantly different from saline (* *p*<0.05, two-way ANOVA, Bonferroni's *post hoc* test). Values are mean ±SEM.

The effect of the F639A mutation on the anxiolytic properties of ethanol was tested using an elevated zero maze. There were no genotypic specific differences in the percentage of time spent in the open arm of the maze between saline-treated F639A Het and wild-type mice (Figure 6A). Treatment with ethanol (1.25 g/kg) increased the percent of time spent in the open arm (two-way ANOVA: effect of treatment, F_(1,36)_ = 10.51, *p*<0.01), but post-hoc analysis showed that this effect was significant only in wild-type mice (*p*<0.01). Ethanol treatment increased locomotor activity (two-way ANOVA: effect of treatment, F_(1,36)_ = 4.67, *p*<0.05; Figure 6B) and total arm entries in both groups, although the latter measure did not quite reach statistical significance (*p* = 0.06; Figure 6C). No genotypic differences in total number of arm entries and total distance traveled were observed in either saline- or ethanol-treated wild-type and F639A Het mice.

### Ethanol Drinking Studies

**Figure 7:**
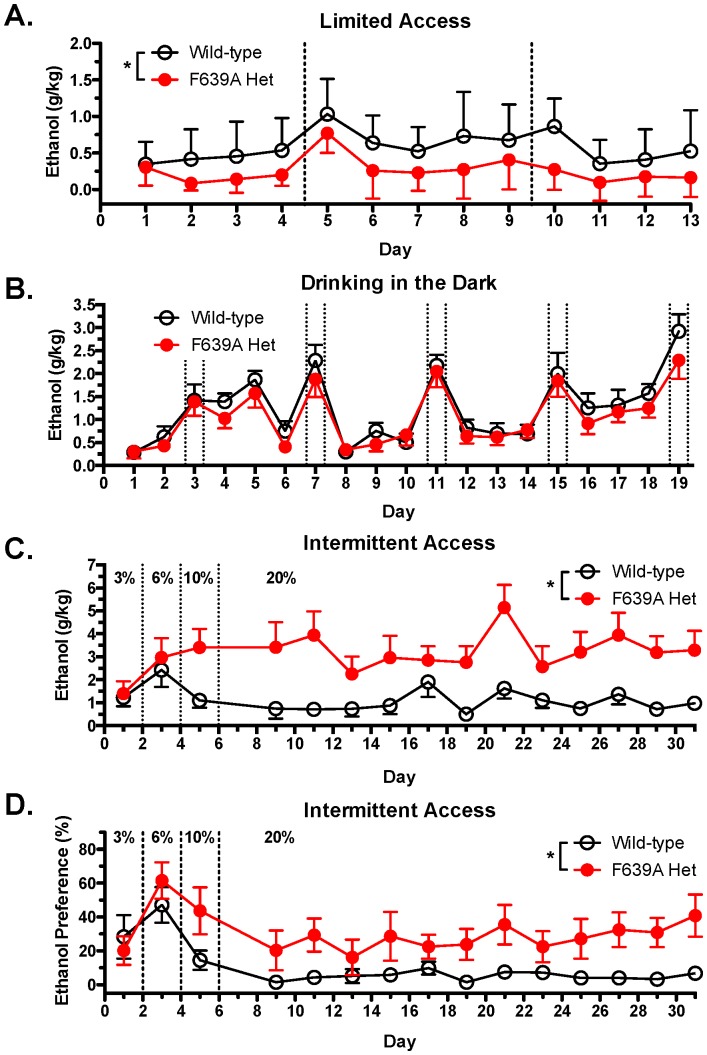
F639A Het mice show altered ethanol consumption than wild-type mice in short-access and long-access drinking paradigms. (*A*), Ethanol intake (mean ±SEM) in wild-type and F639A Het mice during 2 h limited-access to 15% (v/v) ethanol or water (n = 8 for each group). Symbol (*): indicates main effect of genotype (* *p*<0.05, mixed ANOVA). (*B*), Ethanol intake (mean ±SEM) in a limited-access DID model in wild-type and F639A Het mice. Mice had access to one bottle containing 20% (v/v) ethanol 3 h into their dark cycle for 2 h and 4 h sessions (n = 11–12 for each group). Dotted lines indicate 4 h sessions. (*C*), Ethanol intake (mean ±SEM) in wild-type and F639A Het mice during intermittent 24 h access to ethanol or water (n = 10–11 for each group). Ethanol concentrations were ramped from 3, 6, 10% and maintained at 20% (v/v) ethanol. Symbol (*): indicates main effect of genotype (* *p*<0.05, mixed ANOVA). (*D*), Percent preference for ethanol solution over water-bottle choice in a subset of animals from intermittent access study (n = 6 from each group). Symbol (*): indicates main effect of genotype (* *p*<0.05, mixed ANOVA). Values are mean ±SEM.

A variety of well-characterized paradigms were used to test whether the F639A mutation altered ethanol drinking patterns and taste preference in mice. Under limited-access conditions where animals were provided drinking tubes containing 15% ethanol or water for 2 h each day, wild-type mice drank significantly more than F639A Het mice (mixed ANOVA: effect of genotype, F_(1,30)_ = 13.04, *p*<0.001; effect of day, F_(12,117)_ = 6.06, *p*<0.0001; Figure 7A). Water consumption (mls) during the short access period was negligible for both genotypes (wild-type, 0.04±0.02; F639A Het, 0.05±0.02). The genotypic difference in ethanol consumption, while highly significant, was characterized by low amounts of drinking in both groups as 2 h ethanol intake (mean ±SEM; g/kg) during the last five days of drinking was 0.57±0.04 for wild-type mice and 0.22±0.07 for Het mice. This may reflect the mixed background of these animals as 129/S mice show an ethanol consumption that is intermediate between high drinking (C57/Bl6J) and low drinking (DBA2) strains [33]. To increase drinking, a separate cohort of mice were used in the DID paradigm (Figure 7B) that incorporates normal diurnal fluctuations in activity and slightly longer drinking sessions (4 every 4^th^ day) to boost volumes of ethanol consumed. Levels of drinking in the DID model were increased and average ethanol intake (g/kg) during last three sessions of 2 h access was 1.11±0.06 for wild-type mice and 1.38±0.06 in Het mice. Analysis of the DID data revealed no significant effect of genotype on the amount of ethanol consumed although as expected, there was an effect of session length on intake (mixed ANOVA: effect of day, F_(18,219)_ = 18.55, *p*<0.0001; effect of session length, F_(1,316)_ = 227.59, *p*<0.0001).

**Figure 8:**
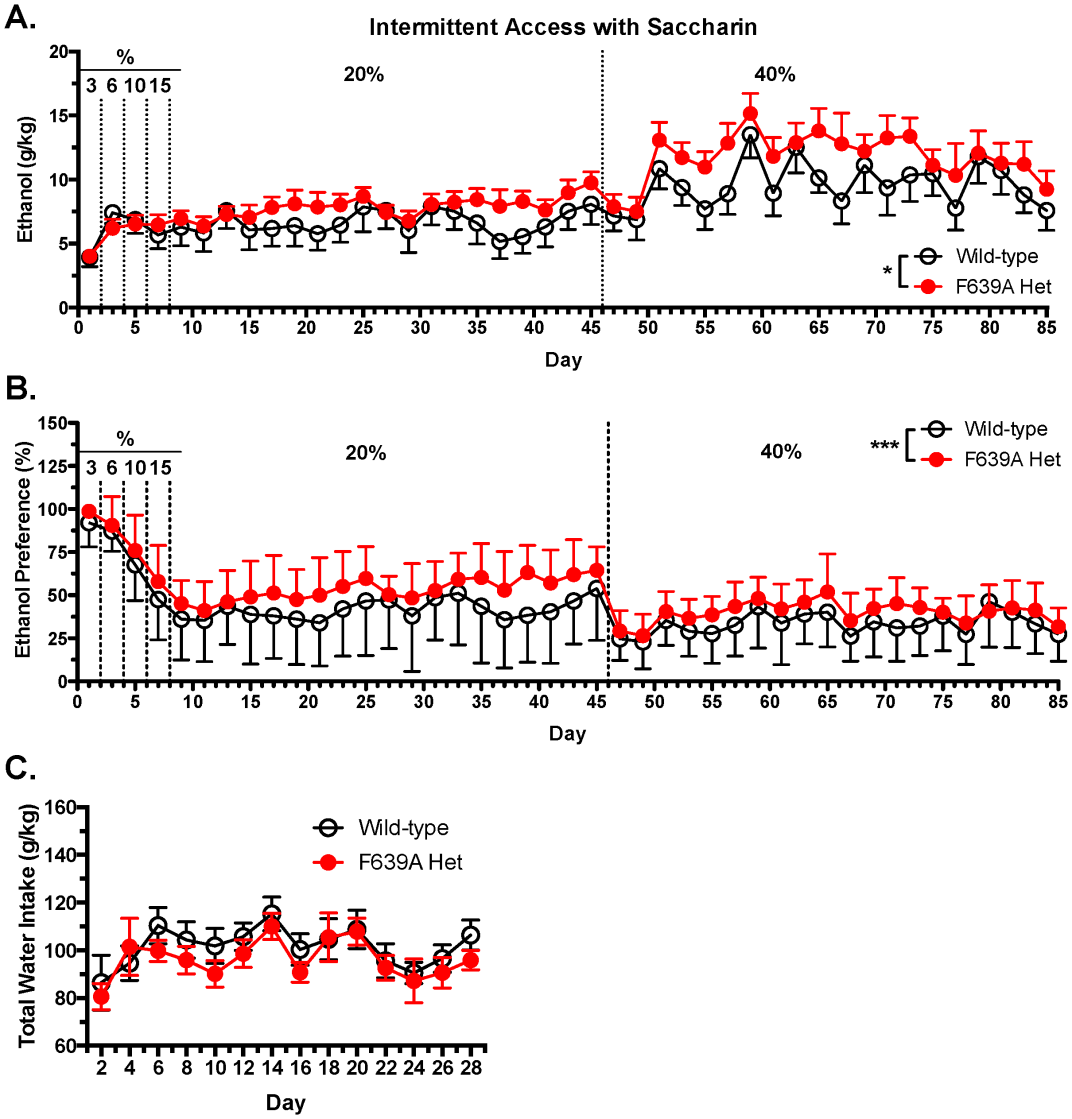
F639A Het mice consume more of a sweetened ethanol solution than wild-type mice in long-access drinking paradigm. (*A*), Ethanol intake (mean ±SEM) in wild-type and F639A Het mice with intermittent 24 h access to sweetened ethanol or water (n = 10–11 for each group). Ethanol concentrations were ramped from 3–20% (v/v) and all concentrations also contained 0.2% saccharin (w/v). Symbol (*): indicates main effect of genotype (* *p*<0.05, mixed ANOVA). (*B*), Percent preference for sweetened ethanol solution over water. Symbol (*): indicates main effect of genotype (*** *p*<0.001, mixed ANOVA). Values are mean ±SEM. (*C*), Total water intake (mean ±SEM) during ‘off’ drinking days in which mice received 2 bottles containing water.

In contrast to results obtained with the limited-access procedures, F639A Het mice given 24 h access to ethanol every other day (intermittent access; IA) drank significantly more ethanol than wild-type mice as ethanol concentrations exceeded 6% (mixed ANOVA: effect of genotype, F_(1,29)_ = 6.55, *p*<0.05; Figure 7C). F639A Het mice also demonstrated an overall higher preference for ethanol solutions over water than their wild-type littermates (mixed ANOVA: effect of genotype, F_(1,18)_ = 8.02, *p*<0.05; effect of day, F_(11,84)_ = 1.19, *p*<0.05; Figure 7D). The increase in drinking by F639A mice was confirmed in a second IA drinking study using a different cohort of mice that received water or ethanol sweetened with 0.2% saccharin to further boost consumption. In this study, F639A Het mice also consumed more ethanol than wild-type mice when ethanol concentrations increased past 10% and this increase was maintained even when mice were provided with a 40% sweetened ethanol solution (mixed ANOVA: effect of genotype, F_(1,70)_ = 5.17, *p*<0.05; effect of day, F_(38,402)_ = 3.92, *p*<0.0001; Figure 8A). F639A Het also displayed higher preference for the sweetened ethanol solution over water compared to wild-type mice (mixed ANOVA: effect of genotype, F_(1,70)_ = 8.97, *p*<0.0001; effect of day, F_(38, 411)_ = 4.511, *p*<0.0001; Figure 8B). There were no genotypic differences in water consumption on the intermittent water days (mixed ANOVA: effect of genotype, F_(1,19)_ = 0.47, *p* = 0.5; effect of day, F_(14,174)_ = 14.43, *p*<0.0001; Figure 8C).

### Tastant Testing and Ethanol Aversion

**Figure 9:**
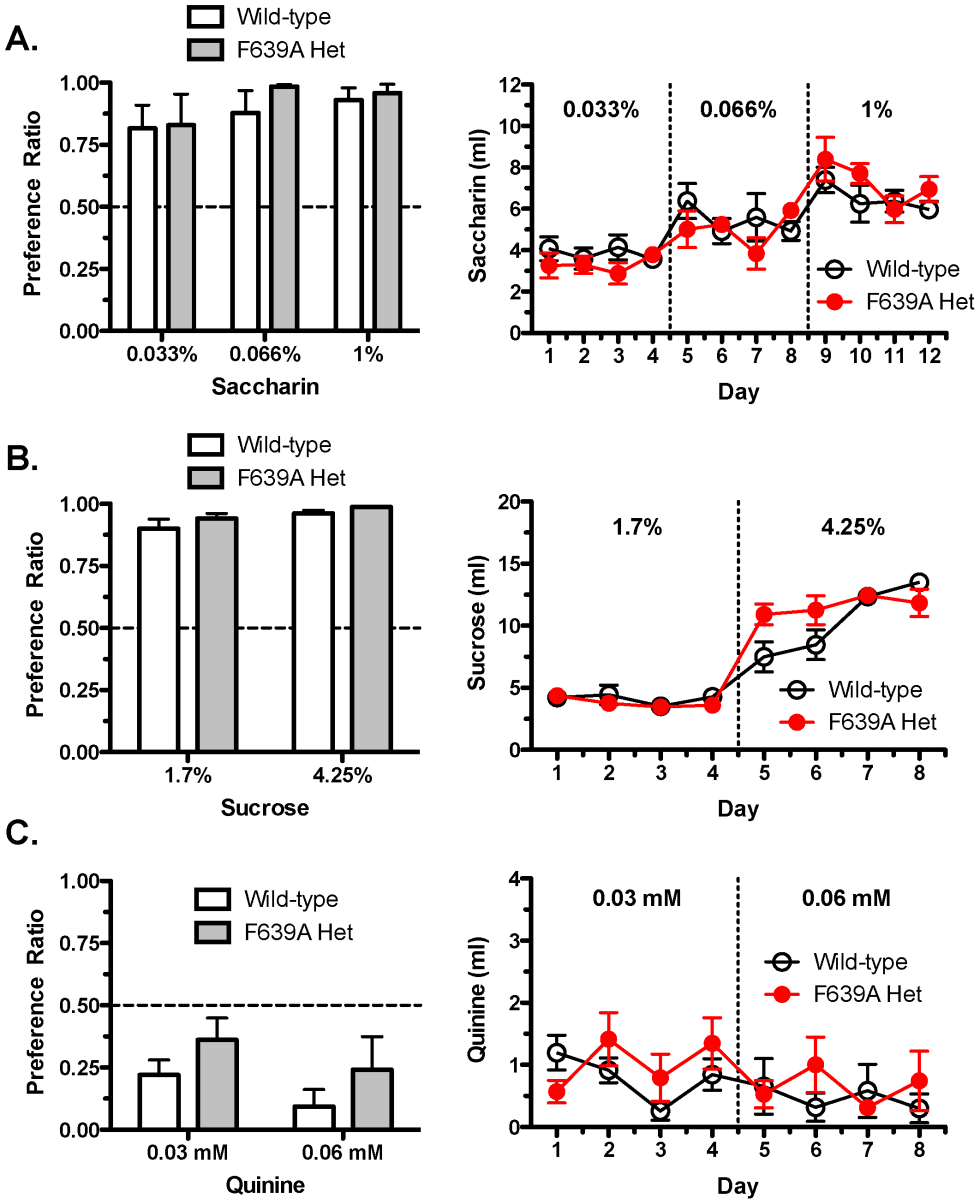
F639A Het and wild-type mice do not differ in taste reactivity. Consumption in wild-type and F639A Het mice was measured using a two-bottle choice test with 24 h continuous access to tastants (n = 7 for each group). Left panels show preference ratio for volume of tastant solution consumed over water measured on the 4^th^ day of access for (*A*) saccharin, (*B*) sucrose, and (*C*) quinine. Right panels show corresponding volumes consumed across days for each tastant. Values are mean ±SEM.

Differences in ethanol consumption between wild-type and F639A Het mice might reflect fundamental differences in taste perception or sensation. To test this, preference ratios and total volume consumption for sweet and bitter tastants were determined in wild-type and F639A Het mice using a two-bottle choice continuous-access paradigm. There were no significant genotypic differences between groups of mice in their preference for saccharin (two-way RM ANOVA: effect of genotype, F_(1,12)_ = 0.30, *p* = 0.60; Figure 9A), sucrose (two-way RM ANOVA: effect of genotype, F_(1,12)_ = 1.50, *p* = 0.24; Figure 9B), or quinine (two-way RM ANOVA: effect of genotype, F_(1,12)_ = 1.37, *p* = 0.27; Figure 9C). Preference for sucrose and quinine depended on concentration of tastant (two-way RM ANOVA: effect of concentration, sucrose, F_(1,12)_ = 10.61, *p*<0.01; quinine, F_(1,12)_ = 11.47, *p*<0.01). There was also no genotypic difference in the volume of each tastant consumed across days between F639A Het and wild-type mice although a day × genotype interaction was seen for sucrose (two-way RM ANOVA: effect of day, sucrose, F_(7,84)_ = 88.06, *p*<0.0001; saccharin, F_(11,132)_ = 14.31, *p*<0.0001; day × genotype interaction, sucrose, F_(7,84)_ = 4.35, *p*<0.0001).

**Figure 10:**
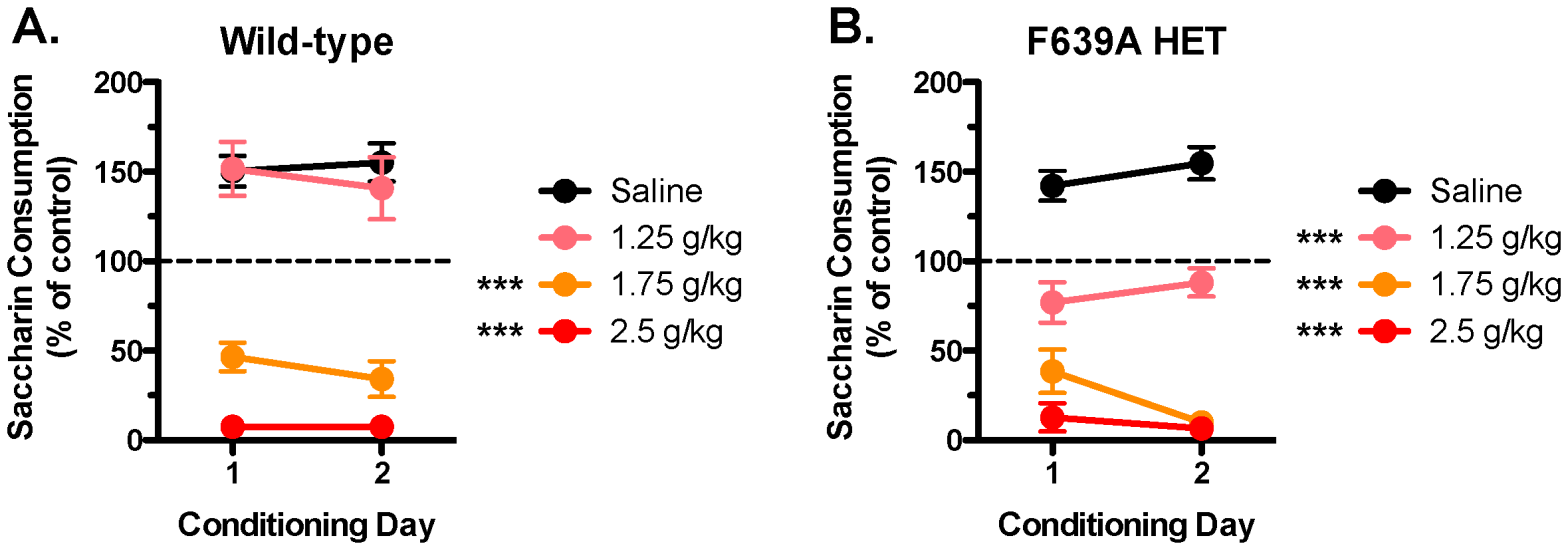
F639A Het mice show altered conditioned taste aversion to a low dose of ethanol as compared to wild-type mice. Graphs show percent of baseline saccharin solution consumed after repeated pairings with an injection of saline, 1.25/kg, 1.75 g/kg, or 2.5 g/kg of ethanol in (*A*) wild-type, and (*B*) F639A Het mice (n = 6–7 for each group). Symbol (*): value significantly different from saline (*** *p*<0.001, two-way RM ANOVA, Bonferroni's *post hoc* test). Values are mean ±SEM.

We used a conditioned taste aversion learning assay to investigate any potential genotypic differences in the aversive effects of ethanol [34]. Animals given daily injections of saline following consumption of a sweetened (0.15% saccharin) solution showed no taste aversion and both groups of mice increased the volume of saccharin consumed across saline-conditioning days (Figure 10). Wild-type mice conditioned with a low/moderate dose of ethanol (1.25 g/kg) showed the same escalation in saccharin consumption seen in saline-treated mice while F639A Het mice showed no change in consumption from the pre-conditioning session. Both groups showed robust taste aversion when saccharin consumption was paired with an injection of 1.75 g/kg or 2.5 g/kg ethanol (two-way RM ANOVA: effect of treatment, wild-type, F_(3,23)_ = 60.50, *p*<0.0001; F639A Het, F_(3,23)_ = 78.08, *p*<0.0001; treatment × conditioning day interaction, F639A Het, F_(1,23)_ = 4.181, *p*<0.05).

## Discussion

In this study, knock-in mice expressing a mutant GluN1 subunit that reduces ethanol inhibition of NMDARs showed task-specific alterations in their responses to alcohol as compared to wild-type littermates. Locomotor activity in GluN1(F639A) knock-in mice was not enhanced by low doses of ethanol and mutant mice recovered faster from motor incoordination than control animals following higher dose ethanol administration. Knock-in mice also showed a reduced anxiolytic response to ethanol and altered patterns of ethanol consumption. There were no differences in the sedative-hypnotic or hypothermic effects of ethanol between wild-type and mutant mice and both genotypes had similar taste reactivity and rates of alcohol metabolism. Overall, these findings provide the most direct evidence to date to support the long-standing hypothesis that NMDARs are key mediators of the behavioral actions of ethanol.

Both pharmacological and genetic approaches have been used to investigate the link between NMDARs and ethanol-induced behaviors. Mice pretreated with NMDA antagonists such as MK-801 or phencyclidine show enhanced ethanol-induced sleep time and motor impairment but no change in ethanol-induced hypothermia [35]. In contrast, pretreatment of mice with the GluN2B antagonist Ro-25-6891 had little effect on ethanol-induced sleep time except at the highest dose tested [36]. Using receptor co-agonists, Lockridge *et al* [37] showed that pretreatment with the GluN1 agonist D-serine increased the latency to ethanol-induced LORR and reduced sleep time in mice. These manipulations had no effect on ethanol-induced impairment of rotarod performance but did reduce ethanol drinking although only under a free-choice paradigm. Debrouse *et al* [38] also showed no effect of D-serine pretreatment on ethanol-induced ataxia but did not find that D-serine reduced ethanol-induced hypnosis. This discrepancy could reflect differences in pretreatment interval as D-serine applied with or after ethanol injection had no effect on these responses [37]. D-serine also appeared to increase ethanol metabolism and prevented the decrease in serum levels of L-serine following ethanol injection [37]. These results highlight the potential problems in using pharmacological agents to probe ethanol action in vivo as various off-target effects are often not easily identified or controlled for.

Knockout mice lacking GluR1 or GluN2A subunits show normal loss of righting reflex and sleep time following high dose ethanol administration [35]. These mice also had the normal potentiation in ethanol-induced ataxia and sleep time following MK-801 injection. Although ethanol's anxiolytic and anti-depressant actions were not tested in these mice, GluN2A KO mice show reduced baseline levels of anxiety and depression-related behaviors supporting a role for NMDA receptors in emotional processing [39]–[41].

Unlike GluN2A null mice, germline deletion of either GluN1 or GluN2B subunits is lethal [30], [42]. Badanich *et al* [43] circumvented this problem by using a Cre-mediated conditional KO mouse to reduce GluN2B expression in forebrain, dorsal and ventral striatum, amygdala and BNST of adult mice. These mice had higher basal levels of locomotor activity that was further enhanced following low dose ethanol. GluN2B null mice were hypersensitive to the locomotor depressant effects of ethanol and slept longer than wild-type mice following high dose ethanol. These results are somewhat counter-intuitive as NMDA-mediated electrophysiological responses in these mice were reported to be essentially insensitive to ethanol [44]. Badanich *et al* [43] suggested that deletion of GluN2B containing NMDARs may have altered the normal network and signal transduction processes that regulate the motor and sedative effects of ethanol thus making these animals hypersensitive to alcohol. A similar enhancement in ethanol sedation was reported for mice lacking PSD-95, a protein highly expressed in glutamatergic synapses [45]. As both GluN2B and PSD-95 are critical regulators of much of the plasticity of glutamatergic synapses, loss of either one of these proteins may destabilize synapses and lead to altered sensitivity to acute ethanol as well as impairments in mechanisms that underlie rapid tolerance to ethanol [46]–[48].

A major finding of the present study is the differential effect that the GluN1(F639A) mutation had on voluntary ethanol consumption. Under limited-access conditions that produce relatively low ethanol consumption, mutant mice drank less than their wild-type counterparts. This was probably not due to altered taste sensation or metabolism as mutant mice showed no differences in preference for sweet or bitter substances or alcohol clearance. Instead, this change may reflect a dampening of ethanol's rewarding effects in mutant mice due to their lack of sensitivity to lower concentrations of ethanol associated with limited-access drinking. Ethanol and other drugs of abuse are thought to produce reward by enhancing the release of dopamine from neurons in the ventral tegmental area [49]. The mechanisms underlying this effect are complex and likely drug-specific but for ethanol may involve inhibition of NMDA receptors as highly selective inhibitors of NMDARs such as MK-801, PCP and ketamine all enhance dopamine release in reward-associated areas such as nucleus accumbens and prefrontal cortex that receive projections from VTA DA neurons [50]–[52]. Anatomical studies reveal that PFC neurons synapse onto mesocortical but not mesolimbic VTA DA neurons and also make extensive contacts with GABAergic interneurons within the VTA and onto GABA projection neurons that innervate the nucleus accumbens [53]. PFC output could thus promote or inhibit VTA dopamine activity based on whether individual DA neurons project to cortical or limbic areas. In a recent study from this lab, pharmacological manipulation of PFC activity inversely regulated changes in mesolimbic VTA DA neuron plasticity following a brief exposure to the abused inhalant toluene [54]. In light of these findings, we hypothesize that, in wild-type mice, the local excitatory action of ethanol on DA neurons [55]–[57] is enhanced by ethanol inhibition of NMDA receptors on PFC neurons that provide top-down control of VTA DA neuron excitability. Due to expression of NMDARs with decreased ethanol sensitivity, this PFC-dependent inhibition of DA neurons would persist longer in GluN1(F639A) mice particularly when drinking periods are short. Paradoxically, in the intermittent access model with longer drinking periods, mutant mice drank more than wild-type animals and this was apparent even at ethanol concentrations as high as 40%. These results imply that GluN1(F639A) mice have a higher reward threshold than wild-type mice and thus may need to drink more to fully engage reward-related mechanisms. An alternative hypothesis to explain these findings suggests that mutant mice may lack the normal “stop” signal that curtails drinking when access is not limited [58], [59]. In mice and humans, the degree of ethanol inhibition of NMDARs may be one signal that prompts most individuals to stop drinking before severe aversive or unpleasant feelings arise. As NMDA-mediated EPSCs in mutant mice were clearly less inhibited by intoxicating concentrations of ethanol as compared to wild-type mice, the amount of ethanol required to reach an aversive set-point would be increased thus leading to higher levels of drinking. In the taste aversion studies, higher doses of ethanol (1.75, 2.5 g/kg) produced a robust inhibition of saccharin drinking in both wild-type and F639A Het mice, while a lower dose (1.25 g/kg) reduced drinking only in F639A mice. While these findings initially appear at odds with the stop-signal hypothesis described above, aversion to drugs like ethanol may arise from the novelty of the subjective intoxication rather than from toxicity [34]. Thus, the aversive effects of ethanol in wild-type mice may be countered by its anxiolytic action especially at lower doses while the lack of such effect in F639A mice may promote aversion and reduced drinking upon subsequent presentation of the sweetened solution.

While the results discussed above suggest an important role for NMDA receptors in mediating selective actions of ethanol, there are several important caveats to be considered. First, all studies were conducted with mice backcrossed with C57Bl/6J mice for two generations and these mice may have retained genes from the parent 129S1/X1 parental background that are linked to the targeted locus that could influence ethanol consumption and other effects of ethanol. Secondly, the studies with adult animals used mice heterozygous for the modified *Grin1* allele due to the unexpected lethality of neonatal homozygous individuals. This lethality is unlikely to reflect insufficient NMDA expression or function as currents in neurons cultured from embryonic homozygous mice were similar to those of wild-type and heterozygous counterparts. Our previous findings show that while the F639A mutation reduced ethanol inhibition of all recombinant receptors tested, this was accompanied by a small but significant leftward shift in the glycine [21] but not glutamate [60] dose response curve. Basal levels of glycine and/or D-serine in the brain are normally sufficient to support NMDA receptor activity, though a heightened sensitivity to co-agonist as implied by the faster response to bath applied NMDA in neurons from knock-in mice might result in abnormal receptor function especially during the critical post-natal period. Unidentified compensatory changes in neuronal function in homozygous mutant mice could also contribute to their neonatal lethality although such changes appear to be normalized by the presence of the wild-type GluN1 subunit as heterozygous animals were viable, grew and bred normally, and had normal levels of GluN1 and GluN2 subunit expression in most brain regions tested. Nonetheless, although the observed changes in ethanol-induced behaviors in mutant mice likely result from the reduced ethanol inhibition of F639A containing NMDARs, we can not rule out the possibility that these effects may be due to alterations in receptor function that are secondary to the change in ethanol sensitivity.

With the above caveats in mind, the results of the present study support the idea that NMDARs are important in mediating selective actions of ethanol including drinking. These findings are relevant to the understanding of the underlying causes of alcohol dependence and support studies that have linked the sensitivity of individuals with a positive family history (FH+) of alcoholism to selective NMDA antagonists. FH+ subjects report reduced feelings of intoxication following administration of low doses of ketamine [61], [62] and ethanol [63] and this shift in sensitivity may contribute to the escalation in alcohol consumption commonly observed in patients with a family history of alcohol dependence. Determining what factors regulate the acute ethanol sensitivity of NMDARs may reveal novel treatments that can reduce the risk of developing alcohol use disorders among susceptible individuals.
